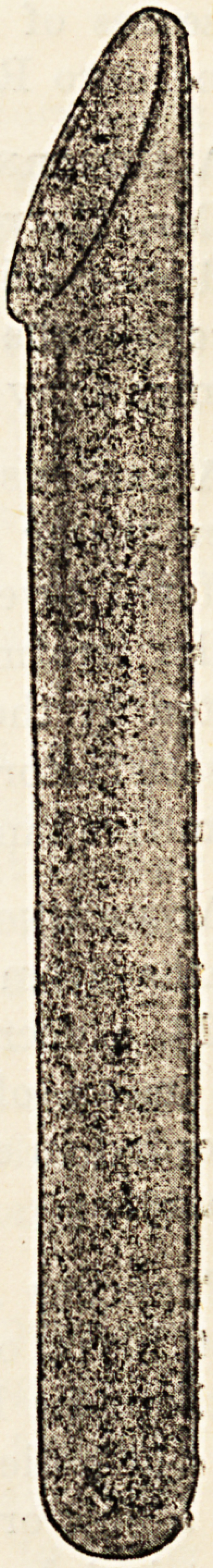# New Appliances & Things Medical

**Published:** 1909-04-24

**Authors:** 


### NEW APPLIANCES & THINGS MEDICAL.

### THE IMPROVED TEST TUBE.

We have received from Messrs. Baird and Tatlock, scientific instrument makers and laboratory furnishers, of 45 Renfrew Street, Glasgow, a new form of test tube devised by Dr. Wyllie Nicol, of the Glasgow Hospital for Skin Diseases. It is suitable for all tests, and is specially convenient for those in which it is necessary to add fluids gently and gradually, as in the nitric acid test for albumen. The large oblique mouth allows of the tube being held at any angle. Reagent bottles, even when not very suitable in shape, can be quickly adjusted to it. Pouring and dropping in are easily controlled, and drop bottles can be used with the tube at any angle. With the rim at the upper end turned in there is little risk of soiling. The tube can be readily closed with the thumb, for which the size and shape of the opening are well suited. It can be cleaned as easily as the ordinary type of test tube. The size and shape of the mouth and rim may be varied to suit special requirements; but the form shown in the illustration is the most useful. The tube may be had in all sizes. This simple improvement of the ordinary test tube is distinctly ingenious, and we can recommend it.

## Figures and Tables

**Figure f1:**